# Antimicrobial resistance, extended-spectrum β-lactamase determinants, and virulence gene profiles of *Escherichia coli* along the pork production chain in central Thailand

**DOI:** 10.14202/vetworld.2026.52-64

**Published:** 2026-01-08

**Authors:** Watsawan Prapasawat, Achiraya Siriphap, Sirikarn Wiriyasirivaj, Apiradee Intarapuk, Ruttana Pachanon, Chie Nakajima, Yasuhiko Suzuki, Orasa Suthienkul

**Affiliations:** 1 Department of Clinic, Faculty of Veterinary Medicine, Mahanakorn University of Technology, Bangkok 10530, Thailand; 2 Division of Microbiology and Parasitology, School of Medical Sciences, University of Phayao, Phayao 56000, Thailand; 3 Department of Science, Technology and Innovation, Faculty of Science, Chulabhorn Royal Academy, Bangkok, Thailand; 4 Division of Bioresources, Hokkaido University International Institute for Zoonosis Control, Sapporo, Japan; 5 International Collaboration Unit, Hokkaido University International Institute for Zoonosis Control, Sapporo, Japan; 6 Hokkaido University Institute for Vaccine Research and Development, Sapporo, Japan; 7 Faculty of Public Health, Thammasat University (Rangsit Campus), Pathumthani 12121, Thailand; 8 Faculty of Public Health, Mahidol University, Bangkok 10400, Thailand

**Keywords:** antimicrobial resistance, extended-spectrum beta-lactamase, *Escherichia coli*, food safety, multidrug resistance, pork production chain, slaughterhouse contamination, Thailand, virulence genes

## Abstract

**Background and Aim:**

Antimicrobial resistance (AMR) in foodborne bacteria presents a significant threat to public health, especially in countries with intensive livestock production systems. Pig farming is a major source of animal protein in Thailand and is recognized as an important reservoir of antimicrobial-resistant bacteria. *Escherichia coli* is commonly used as an indicator organism for monitoring AMR, including extended-spectrum β-lactamase (ESBL) production and pathogenic potential. This study aimed to assess the frequency of AMR, multidrug-resistant (MDR), ESBL determinants, and virulence genes in *E. coli* isolates collected from slaughterhouses and fresh markets in central Thailand.

**Materials and Methods:**

A total of 498 archived *E. coli* isolates were analyzed, including 236 isolates from slaughterhouses (feces and carcasses) and 262 isolates from fresh markets (pork and cutting boards). Antimicrobial susceptibility testing was performed against 18 antimicrobial agents using the disk diffusion method. MDR was defined as resistance to three or more antimicrobial classes. ESBL production was identified through phenotypic confirmatory tests, and ESBL-producing isolates were screened for *bla*_TEM_, *bla*_CTX-M_, and *bla*_SHV_ genes by multiplex polymerase chain reaction. All isolates were further examined for select virulence genes linked to major *E. coli* pathotypes.

**Results:**

Overall, 97.4% of *E. coli* isolates showed resistance to at least one antimicrobial agent, and 87.3% were classified as MDR. ESBL-producing *E. coli* made up 23.5% of all isolates, with a significantly higher prevalence in slaughterhouses compared to fresh markets (p < 0.05). Among ESBL producers, 97.4% exhibited MDR phenotypes. Most (89.7%) of the ESBL-producing isolates carried at least one *bla* gene, with *bla*_TEM_ being the most common, followed by *bla*_CTX-M_. Virulence genes were detected at a low frequency (3.2%), mainly involving *eaeA*, *lt*, and *stp*.

**Conclusion:**

The high prevalence of AMR, MDR, and ESBL-producing *E. coli* throughout the pork production chain highlights slaughterhouses and fresh markets as key points for the spread of resistant bacteria. These findings emphasize the need for stronger antimicrobial stewardship, better hygiene practices, and ongoing AMR surveillance within the One Health approach to reduce public health risks linked to pork consumption.

## INTRODUCTION

Antimicrobial resistance (AMR) is widely acknowledged as a major global public health threat, causing approximately 700,000 deaths worldwide each year [1]. The widespread use of antimicrobial agents in both human healthcare and animal production systems is a key factor in the emergence and spread of AMR. In Thailand, pigs are one of the main food-producing animals and have been identified as a significant reservoir of antimicrobial-resistant bacteria [2, 3]. In pig production systems, antimicrobials are commonly used for both therapeutic and growth-promotion purposes. Previous studies have reported that about 78%–99% of *Escherichia coli* isolates from pigs show AMR phenotypes [4]. Antimicrobial-resistant bacteria colonizing food animals can be transmitted to humans through the consumption and handling of contaminated food products, direct contact with animals, and environmental spread of resistant organisms [2].

Additionally, a rising trend in the emergence of multidrug-resistant (MDR) bacteria in food animals has been observed [5]. Numerous studies have emphasized the increasing prevalence of extended-spectrum β-lactamase (ESBL)-producing *E. coli*, which represents a global public health crisis. ESBLs are enzymes that provide resistance to extended-spectrum cephalosporins, such as cefotaxime, ceftazidime, and ceftriaxone, as well as to monobactams [6]. These enzymes have been increasingly detected among members of the *Enterobacteriaceae* family, especially *E. coli* strains isolated from food-producing animals [4]. The most commonly identified ESBL-associated genes include *bla*_CTX_ and *bla*_TEM_. Additionally, various ESBL genes have been found in *E. coli* from diverse animal-derived food sources [7]. These reservoirs are particularly concerning because they may harbor *E. coli* strains resistant to critically important antimicrobials and help facilitate their transmission to humans via the food chain.

*E. coli* is also the etiological agent of colibacillosis, a common infectious disease affecting both pigs and humans [8]. In pigs, colibacillosis poses a significant challenge to the industry due to its association with increased morbidity and mortality [9]. Enterotoxigenic *E. coli* (ETEC), enteropathogenic *E. coli* (EPEC), and Shiga toxin–producing *E. coli* (STEC) are the main causes of neonatal diarrhea, post-weaning diarrhea, and edema disease in pigs [10]. These pathotypes are not only economically important in pig production but also pose significant foodborne public health risks, having been linked to severe and sometimes deadly outbreaks worldwide. As a result, pigs are widely recognized as important reservoirs of pathogenic *E. coli*, with the potential to contaminate pork products and transmit infections to consumers [8].

In Thailand, pig production mainly occurs in the central provinces around Bangkok, which together make up about 36%–40% of the country’s total pig output [11].

Despite growing recognition of AMR and ESBL-producing *E. coli* as major threats to food safety and public health, significant knowledge gaps remain along the pork production chain in Thailand. Most existing studies have focused on farm-level surveillance or retail meat products, with limited integration of multiple critical points within the same production process. In particular, systematic data linking slaughterhouse environments (fecal and carcass contamination) with downstream fresh-market settings (pork and food-contact surfaces such as cutting boards) are scarce. This lack of integrated surveillance hampers the ability to identify key contamination points and understand how antimicrobial-resistant *E. coli* persist and spread from primary processing to retail environments.

Furthermore, while several studies have reported the prevalence of AMR or ESBL-producing *E. coli* in food animals, fewer investigations have simultaneously characterized MDR patterns, ESBL determinants, and virulence gene profiles within the same isolate collection. Such combined analyses are crucial to distinguish between commensal reservoirs of resistance and strains with potential pathogenicity. In addition, region-specific data from central Thailand, where pig production and pork distribution are highly concentrated, remain limited, especially concerning the comparative burden of resistance and ESBL genes between slaughterhouses and fresh markets. The lack of this information limits evidence-based risk assessment and the development of targeted intervention strategies across the pork production chain.

This study aimed to thoroughly examine AMR, MDR, ESBL production, and virulence gene profiles of *E. coli* strains collected from key points along the pork production chain in central Thailand. Specifically, it sought to (i) identify AMR patterns and the prevalence of MDR in *E. coli* isolates from slaughterhouses and fresh markets; (ii) determine the frequency of ESBL-producing *E. coli* and analyze the distribution of major ESBL-related genes (*bla*_TEM_*, bla*_CTX-M_, and *bla*_SHV_); (iii) investigate the presence of specific virulence genes linked to clinically and epidemiologically significant *E. coli* pathotypes; and (iv) compare resistance profiles and gene distribution between the upstream (slaughterhouse) and downstream (fresh-market) stages of the pork supply chain. By achieving these objectives, the study aims to provide baseline data to support risk-reduction strategies, antimicrobial stewardship, and enhanced food-safety measures within a One Health approach.

## MATERIALS AND METHODS

### Ethical approval

This study did not involve live animals or human participants. All *E. coli* isolates analyzed were obtained from a preserved stock culture collection maintained by the Faculty of Public Health at Thammasat University, Thailand, which were originally collected during routine microbiological surveillance activities conducted in 2017–2018. No animals were handled, restrained, sampled, or subjected to any intervention by the research team. According to Thai national regulations, studies using archived bacterial isolates do not require approval from an Institutional Animal Care and Use Committee or a Human Research Ethics Committee. Institutional exemption was granted by the Ethics Review Committee of the Faculty of Public Health at Thammasat University, confirming that the study was exempt from ethical review because it involved only preserved microorganisms and no identifiable animal, human, or personal data (Exemption ID: 0516.71/706). All laboratory procedures and data handling followed institutional biosafety guidelines, Thailand Ministry of Public Health regulations, and international standards for research involving microbial isolates.

### Study design, period, and location

This study used a retrospective laboratory-based design with *E. coli* isolates collected along the pork production chain, including three slaughterhouses and four fresh markets in a central province of Thailand. Sample collection took place during 2017–2018. All *E. coli* isolates were stored at −80°C in glycerol stock cultures at the Faculty of Public Health, Thammasat University. Between 2019 and 2020, representative isolates from the stored stocks were selected for laboratory analysis to assess AMR profiles, ESBL determinants, and virulence genes.

### Origin and selection of *E. coli* isolates

*E. coli* isolates were collected from slaughterhouses supervised by the Department of Livestock Development, Ministry of Agriculture and Cooperatives, and from large fresh markets in a central province of Thailand. All samples were initially identified as *E. coli* using standard bacterial culture techniques and biochemical tests, then stored at –80°C in glycerol. A total of 509 *E. coli* isolates were recovered from the preserved cultures. Of these, 498 isolates were successfully revived and included in the study, while 11 isolates could not be cultured. The analyzed isolates included samples from slaughterhouses (117 fecal and 119 carcass isolates) and from fresh markets (116 pork and 146 cutting board isolates).

### Bacterial identification

All 498 *E. coli* isolates were recovered from preserved stock cultures and verified for purity by streaking onto MacConkey agar (Merck, Darmstadt, Germany), followed by incubation at 37°C for 18–24 h. Typical *E. coli* colonies appeared as pink colonies with a surrounding precipitation zone. Presumptive isolates were biochemically confirmed using the IMViC tests (indole, methyl red, Voges–Proskauer, and citrate) according to the method described by Feng and Weagant [12].

### Antimicrobial susceptibility testing

Antimicrobial susceptibility testing was performed by disk diffusion on Mueller–Hinton agar (MHA; Oxoid Ltd., Basingstoke, United Kingdom) according to the Clinical and Laboratory Standards Institute (CLSI) guidelines [13]. A total of 18 antimicrobial agents were tested, including aminoglycosides (gentamicin, kanamycin, streptomycin), a beta-lactam combination (amoxicillin–clavulanic acid), cephems (cefoxitin, cefotaxime, ceftazidime, cefepime), a folate pathway antagonist (sulfamethoxazole–trimethoprim), a polymyxin (colistin), monobactams (imipenem, meropenem), a nitrofuran (nitrofurantoin), a penicillin (ampicillin), a phenicol (chloramphenicol), quinolones (ciprofloxacin, nalidixic acid), and a tetracycline (tetracycline).

*E. coli* American Type Culture Collection (ATCC) 25922 was used as a quality control strain. Each isolate was cultured on MHA (4 mm depth) and incubated at 37°C for 24 h. Bacterial suspensions were prepared in sterile normal saline (0.85% NaCl) and adjusted to a 0.5 McFarland standard (approximately 10^8^ CFU/mL). The inoculum was evenly spread across MHA plates using sterile cotton swabs, antimicrobial disks were applied, and the plates were incubated at 35°C ± 2°C for 16–18 h. Inhibition zone diameters were measured with a Vernier caliper and interpreted according to CLSI (2020) criteria [13]. MDR was defined as resistance to three or more antimicrobial classes [14].

### Screening for ESBL-producing isolates

ESBL-producing *E. coli* isolates were first identified by disk diffusion screening, in which inhibition zone diameters of ≤22 mm for ceftazidime and ≤27 mm for cefotaxime indicated potential ESBL production according to CLSI (2020) guidelines [13]. Suspected isolates were confirmed using the combination disk method with cefotaxime (30 μg) and ceftazidime (30 μg), tested alone and with clavulanic acid (10 μg) (BD, Franklin Lakes, NJ, USA). An increase of ≥5 mm in the inhibition zone diameter for the antimicrobial agent with clavulanic acid compared to the agent alone confirmed ESBL production. *E. coli* ATCC 25922 was used as the quality control strain.

### DNA extraction

All *E. coli* isolates were cultured in Luria–Bertani broth (Difco, Detroit, MI, USA) at 37°C for 15–18 h. Genomic DNA was extracted using the QIAamp® DNA Mini Kit (Qiagen, Hilden, Germany) according to the manufacturer’s instructions. DNA concentration and purity were measured with a NanoDrop spectrophotometer (Thermo Fisher Scientific, Waltham, MA, USA), and the extracted DNA was stored at −20°C until further use.

### Detection of β-lactamase genes

All ESBL-producing *E. coli* isolates were screened for the presence of β-lactamase genes *bla*_TEM_, *bla*_CTX-M_, and *bla*_SHV_ using multiplex polymerase chain reaction (PCR) assays. Primer sequences are provided in Table 1 [15–17]. Each PCR reaction (20 μL) contained Green GoTaq reaction buffer, deoxynucleoside triphosphates, forward and reverse primers, GoTaq DNA polymerase (Promega, Fitchburg, WI, USA), and template DNA. Thermocycling conditions included an initial denaturation at 94°C for 5 min, followed by 30 cycles of denaturation at 94°C for 30 s, annealing at 60°C for 30 s, and extension at 72°C for 1 min 30 s, with a final extension at 72°C for 10 min. PCR products were separated on 2.0% agarose gels stained with a red stain (Thermo Fisher Scientific, Waltham, MA, USA) and visualized under blue LED illumination using the PrepOne system (Embi Tec, San Diego, CA, USA). Reference strains carrying *bla*_TEM_, *bla*_CTX-M_, and *bla*_SHV_ served as positive controls, while *E. coli* JM109 was used as the negative control.

**Table 1:** Oligonucleotide primers used for polymerase chain reaction amplification.

Target genes	Primer	Sequence (5-3)	Amplicon Size (bp)	References
*bla*_TEM_	TEM-F	TCGGGGAAATGTGCG	1074	[15]
	TEM-R	TGCTTAATCAGTGAGGCACC		
*bla*_SHV_	SHV-F	GCCGGGTTATTCTTATTTGTCGC	1016	[16]
	SHV-R	ATGCCGCCGCCAGTCA		
*bla*_CTX_	CTX-M-uni-F	CGATGTGCAGTACCAGTAA	585	[17]
	CTX-M-uni-R	TAAGTGACCAGAATCAGCGG		
*lt*	LT-F	ATGACGGATATGTTTCCACTTCTC	393	[18]
	LT -R	AACCTTGTGGTGCATGATGAATCC		
*sth*	STh-F	TTCACCTTTCGCTCAGGATGCTA	168	[18]
	STh-R	CACCCGGTACAAGCAGGATT		
*stp*	STp-F	TTAATAACATCCAGCACAGGCAGG	176	[18]
	STp-R	TCCCCTCTTTTAGTCAGTCAACTG		
*stx1A*	stx1A-F2	TCTGCAATAGGTACTCCATTACAG	724	[18]
	stx1A-R2	CCGGACACATAGAAGGAAAC		
*stx2A*	stx2A-F2	TTGACCATCTTCGTCTGATTATTG	542	[18]
	stx2A-R2	CTGATGATGGCAATTCAGTATAAC		
*aggR*	aggRks	GTATACACAAAAGAAGGAAGC	254	[18]
	aggRkas2	ACAGAATCGTCAGCATCAGC		
*pCVD432*	CVD/1	CTCTGGCGAAAGACTGTATC	463	[18]
	CVD/2	CATCTCTACATCAAGAGCAG		
*bfpA*	bfpA-F	AGTCGCAGAATGCTATTTCAGAAG	322	[19]
	bfpA-R	TTTTCGCCAGAGATATTAACACCG		
*eaeA*	eaeA/1a	GCGATTACGCGAAAGATACC	677	[19]
	eaeA/2a	GATAACGGAACTGCATTGAGT		
*ipaH*	ipaH/1	CTGGCTGATGCCGTGACAG	801	[19]
	ipaH/2	GCTGTTCAGTCTCACGCATC		

### Detection of virulence genes

All 498 *E. coli* isolates were tested for 10 virulence genes using multiplex PCR. Primers were arranged into two sets. Primer set A targeted ETEC (*lt*, *sth*, and *stp*), enterohemorrhagic *E. coli* (EHEC) (*stx1A* and *stx2A*), and enteroaggregative *E. coli* (EAEC) (*aggR* and *pCVD432*) [18]. Primer set B targeted EPEC (*bfpA* and *eaeA*) and enteroinvasive *E. coli* (EIEC) (*ipaH*) [19]. Each PCR reaction (25 μL) included Green GoTaq reaction buffer, deoxynucleoside triphosphates, primers, GoTaq DNA polymerase, and template DNA. Amplification conditions featured an initial denaturation at 95°C for 5 min, followed by 35 cycles of denaturation at 95°C for 1 min, annealing at 52°C for 1 min, and extension at 72°C for 1 min, ending with a final extension at 72°C for 5 min. Amplicons were analyzed as described above. Reference strains for each virulence gene served as positive controls, and *E. coli* JM109 was used as a negative control.

### Statistical analysis

Descriptive statistics were used to summarize the frequency and percentage of AMR, ESBL production, and virulence genes among *E. coli* isolates from slaughterhouses and fresh markets. Pearson’s chi-square test and Fisher’s exact test (for expected cell counts <5) were applied to compare detection rates between sample sources, and 95% confidence intervals (CIs) were calculated. Statistical analyses were conducted using the Statistical Package for the Social Sciences (SPSS version 18.0; SPSS Inc., Chicago, IL, USA). A p-value <0.05 was considered statistically significant.

## RESULTS

### AMR phenotypes

The antimicrobial susceptibility of all *E. coli* isolates was assessed across 11 antimicrobial classes using the disk diffusion method with 18 antimicrobial agents. Overall, 97.4% (485/498) of the isolates showed resistance to at least one antimicrobial agent (Table 2). Resistance was detected in 98.3% (232/236) of isolates from slaughterhouses and 96.6% (253/262) from fresh markets. No statistically significant difference in resistance rates was observed between slaughterhouse and fresh-market isolates (p > 0.05; 95% CI: 0.6–6.8).

**Table 2:** AMR detection rate of 498 *Escherichia coli* isolates recovered from slaughterhouses and fresh markets in central, Thailand.

Antimicrobial class	Antimicrobial agents	No. (%) of AMR isolates			

		Slaughterhouses		Fresh markets	
	
		Feces (n = 117)	Carcass (n = 119)	Pork (n = 116)	Cutting board (n = 146)
Resistance		117 (100)	115 (96.6)	116 (100)	137 (93.8)
MDR		110 (94.0)	103 (86.6)	107 (92.2)	115 (78.8)
Aminoglycosides	Gentamicin	43 (36.8)	21 (17.6)	29 (25)	21 (14.4)
	Kanamycin	36 (30.8)	28 (23.5)	17 (14.7)	17 (11.6)
	Streptomycin	71 (60.7)	72 (60.5)	73 (62.9)	76 (52.1)
Phenicols	Chloramphenicol	80 (68.4)	75 (63)	67 (57.8)	73 (50)
Monobactams	Imipenem	1 (0.9)	3 (2.5)	7 (6.0)	3 (2.1)
	Meropenem	0	3 (2.5)	0	1 (0.7)
Cephems	Cefoxitin	1 (0.9)	3 (2.5)	4 (3.4)	3 (2.1)
	Cefotaxime	41 (35)	28 (23.5)	27 (23.3)	20 (13.7)
	Ceftazidime	13 (11.1)	6 (5.0)	6 (5.2)	8 (5.5)
	Cefepime	27 (23.1)	18 (15.1)	14 (12.1)	12 (8.2)
Nitrofurans	Nitrofuratoin	7 (6.0)	5 (4.2)	10 (8.6)	3 (2.1)
Penicillins	Ampicillin	112 (95.7)	109 (91.6)	115 (99.1)	132 (90.4)
β-lactam combination	Amoxicillin/clavulanic acid	11 (9.4)	16 (13.4)	18 (15.5)	0
Polymyxins	Colistin	2 (1.7)	9 (7.6)	7 (6)	5 (3.4)
Quinolones	Ciprofloxacin	25 (21.4)	26 (21.8)	19 (16.4)	10 (6.8)
	Nalidixic acid	52 (44.4)	41 (34.5)	38 (32.8)	30 (20.5)
Folate pathway antagonists	Sulfamethoxazole/ Trimethoprim	62 (53)	66 (55.5)	56 (48.3)	69 (47.3)
Tetracyclines	Tetracycline	98 (83.8)	95 (79.8)	98 (84.5)	100 (68.5)

AMR = Antimicrobial resistance, MDR = Multidrug resistance.

Over 50% of *E. coli* isolates from slaughterhouse samples (feces and carcasses) showed resistance to ampicillin, tetracycline, chloramphenicol, streptomycin, and sulfamethoxazole–trimethoprim. Likewise, isolates from pork and cutting boards in fresh markets exhibited resistance rates over 50% for ampicillin, tetracycline, streptomycin, and chloramphenicol. Conversely, most isolates from both slaughterhouses and fresh markets remained susceptible to imipenem, meropenem, cefoxitin, and colistin.

### MDR and AMR patterns

All 498 *E. coli* isolates were evaluated for MDR based on resistance to various antimicrobial classes. A high rate of MDR was observed, with 87.3% (435/498) of isolates identified as MDR (Table 2). MDR rates were high in both slaughterhouse (90.3%; 213/236) and fresh-market (84.7%; 222/262) isolates. There was no statistically significant difference in MDR prevalence between the two sources (p > 0.05; 95% CI: 0.9–2.8).

A total of 130 unique AMR patterns were identified among all isolates (Supplementary Table 1). The most common patterns were AMI–PHE–PEN–FOL–TET (50 isolates), AMI–PEN–TET (24 isolates), and AMI–PHE–PEN–QUI–FOL–TET (24 isolates).

### Prevalence of ESBL-producing *E. coli*

ESBL-producing *E. coli* accounted for 23.5% (117/498) of all isolates. Of these, 14.3% (71/498) came from slaughterhouses, while 9.2% (46/498) were recovered from fresh markets. The prevalence of ESBL-producing isolates was significantly higher in slaughterhouses than in fresh markets (p < 0.05; 95% CI: 1.3–3.1).

Among slaughterhouse samples, ESBL-producing *E. coli* were found in 34.2% (40/117) of fecal isolates and 26.1% (31/119) of carcass isolates. In fresh markets, ESBL-producing isolates appeared in 18.9% (23/116) of pork samples and 15.8% (23/146) of cutting board samples (Table 3).

**Table 3:** Detection rate of β-lactamase genes in 117 ESBL-producing *Escherichia coli* isolates from slaughterhouses and fresh markets in central Thailand.

Sample types	ESBL-producing *E. coli* (%)	No. (%) of *E. coli* harboring the β-lactamase gene				

		*bla*_TEM_	*bla*_CTX-M_	*bla*_SHV_	*bla*_TEM_ + *bla*_CTX-M_	Not determined
Slaughterhouses	71 (14.3) *	49 (20.8) *	54 (22.9) *	0	34 (14.4) *	0
Feces (n=117)	40	25 (62.5)	34 (85)	0	18 (45)	0
Carcass (n=119)	31	24 (77.4)	20 (64.5)	0	16 (51.6)	3 (9.7)
Fresh markets	46 (9.2) *	29 (11.1) *	15 (5.7) *	2 (0.8)	8 (3.1) *	9 (19.6)
Pork (n=116)	23	13 (56.5)	5 (21.7)	0	2 (8.7)	7 (30.4)
Cutting board (n = 146)	23	16 (69.6)	10 (43.5)	2 (8.7)	6 (26.1)	2 (8.7)
Total	117 (23.5)	78 (66.7)	69 (59)	2 (1.7)	42 (35.9)	12 (10.3)

*E. coli* = *Escherichia coli*, ESBL = Extended-spectrum β-lactamase.

* Significantly different (χ^2^; p < 0.05) *Not determined (none of detected genes).

### MDR among ESBL-producing isolates

Among the 117 ESBL-producing *E. coli* isolates, 97.4% (114/117) were classified as MDR. The prevalence of MDR was similarly high in isolates from slaughterhouses (97.2%; 69/71) and fresh markets (97.8%; 45/46). Among slaughterhouse-derived ESBL producers, MDR was found in 34.2% (40/117) of fecal isolates and 24.8% (29/117) of carcass isolates. In fresh markets, MDR ESBL-producing isolates were present in 19.7% (23/117) of pork samples and 18.8% (22/117) of cutting board samples. Three ESBL-producing isolates were not classified as MDR.

**Figure 1:**
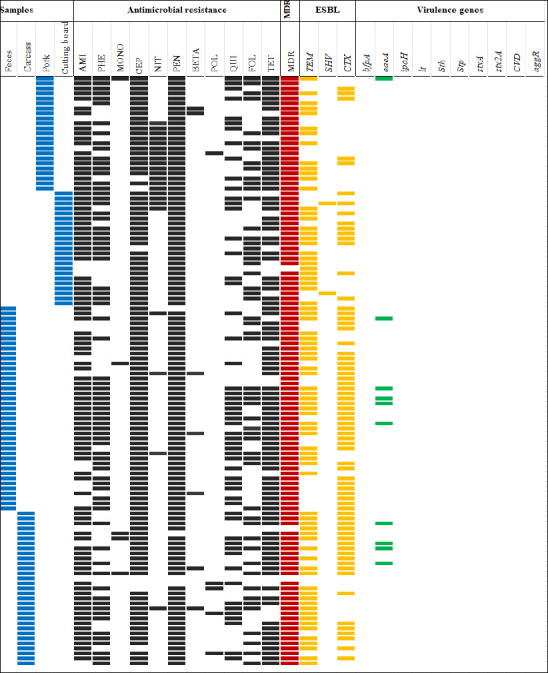
Antimicrobial resistance (AMR) patterns, multidrug resistance (MDR), virulence genes, and β-lactamase genes of 117 ESBL-producing *Escherichia coli* isolates recovered from slaughterhouses and fresh markets in central Thailand. The solid boxes indicate a feature in the isolate. Blue boxes indicate sample types, black boxes indicate AMR, red boxes indicate MDR, yellow boxes indicate ESBL genes, and green boxes indicate virulence genes. AMI = Aminoglycosides, BETA = β-lactam combination, CEP = Cephems, FOL = Folate pathway antagonists, MONO = Monobactams, NIT = Nitrofurans, PEN = Penicillins, PHE = Phenicols, POL = Polymyxins, QUI = Quinolones, TET = Tetracyclines.

A total of 44 unique AMR patterns were identified among ESBL-producing isolates. The most common patterns were AMI–PHE–CEP–PEN–FOL–TET (16 isolates) and AMI–PHE–CEP–PEN–QUI–FOL–TET (16 isolates) (Figure 1).

### Distribution of β-lactamase genes in ESBL-producing isolates

Among the 117 ESBL-producing *E. coli* isolates, 89.7% (105/117) carried at least one of the tested β-lactamase genes (*bla*_TEM_, *bla*_CTX-M_, or *bla*_SHV_). The *bla*_TEM_ gene was the most commonly detected, found in 66.7% (78/117) of isolates, including 69.0% (49/71) from slaughterhouses and 63.0% (29/46) from fresh markets. The *bla*_CTX-M_ gene was present in 59.0% (69/117) of isolates, with a significantly higher prevalence in slaughterhouse isolates (76.0%; 54/71) compared to those from fresh markets (32.6%; 15/46).

The *bla*_SHV_ gene was detected at a low frequency (4.3%; 2/46) and was found only in isolates from fresh markets. Co-occurrence of *bla*_TEM_ and *bla*_CTX-M_ was observed in 35.9% (42/117) of isolates, including 47.9% (34/71) from slaughterhouses and 17.4% (8/46) from fresh markets. The prevalence of *bla*_TEM_, *bla*_CTX-M_, and their combination was significantly higher in slaughterhouse isolates than in fresh-market isolates (p < 0.05) (Table 3).

### Detection of virulence genes in *E. coli* isolates

Virulence genes were found in 3.2% (16/498) of *E. coli* isolates, indicating the presence of pathogenic strains. Three virulence genes, *eaeA*, *lt*, and *stp*, were identified (Table 4). The *eaeA* gene, which encodes intimin and is linked to EPEC and EHEC, was detected in 4.7% (11/236) of isolates from slaughterhouses and 1.1% (3/262) from fresh markets. The *lt* and *stp* genes were each found at a low frequency of 0.8% (2/262) in fresh-market samples, with *lt* identified in one cutting board isolate and *stp* in one pork isolate.

**Table 4:** Detection rate of virulence genes in 498 *Escherichia coli* isolates recovered from slaughterhouses and fresh markets in central Thailand.

Pathotype	Virulence genes	Number (%) of virulence genes			

		Slaughterhouses		Fresh markets	
	
		Feces (n=117)	Carcass (n=119)	Pork (n=116)	Cutting board (n=146)
EPEC/EHEC	*eae*A	5 (4.3)	6 (5.0)	3 (2.6)	0
ETEC	*It*	0	0	0	1 (0.7)
*stp*	0	0	1 (0.9)	0
Total		5 (4.3)	6 (5.0)	4 (3.4)	1 (0.7)

EPEC = Enteropathogenic *Escherichia coli,* EHEC= Enterohemorrhagic *E. coli,* ETEC = Enterotoxigenic *E. coli*

No statistically significant difference was observed in the prevalence of pathogenic *E. coli* between slaughterhouse and fresh-market isolates (p > 0.05; 95% CI: 0.8–7.3). Of the 16 virulence gene–positive isolates, 62.5% (10/16) were MDR and carried β-lactamase genes. The virulence genes *bfpA*, *ipaH*, *sth*, *stx1A*, *stx2A*, *aggR*, and *pCVD432* were not detected in any isolate.

## DISCUSSION

### AMR patterns along the pork production chain

Antimicrobial agents are commonly used in the pork production chain, especially at the farm-level for therapeutic, growth-promoting, and disease-preventing purposes [20]. However, such practices promote the emergence and persistence of AMR at various stages of pork production. In this study, more than 50% of *E. coli* isolates from slaughterhouse samples (feces and carcasses) and fresh-market samples (pork and cutting boards) showed resistance to ampicillin, tetracycline, chloramphenicol, and streptomycin. These antimicrobials are among the ones most frequently linked with resistance in *E. coli* isolates from pigs [21].

The observed resistance patterns align with earlier reports from pig farms in Thailand [4, 22] and slaughterhouse-based studies in the Mekong Delta, Vietnam [23], Brazil [24], and Australia [25]. Notably, studies from Brazil and Australia reported high resistance rates to ampicillin (81.1% and 60.2%, respectively) and tetracycline (97.8% and 68.2%, respectively), along with significant resistance to chloramphenicol [24, 25]. High volumes of tetracyclines, penicillins, and sulfonamides sold for veterinary use have been documented [26], and the improper or excessive use of these antimicrobials in pig production likely drives the elevated resistance rates observed [27]. The detection of chloramphenicol-resistant isolates is especially concerning, as this antimicrobial has been banned for use in food-producing animals [28]. Such resistance may persist due to co-selection or cross-resistance caused by ongoing use of other antimicrobials [29], emphasizing the complex and multifactorial nature of AMR development [28, 30].

Colistin resistance was found in 4.6% of isolates from both slaughterhouse and fresh-market samples. Since colistin is considered a last-resort antibiotic for treating MDR gram-negative infections, this finding raises serious public health concerns. The plasmid-mediated *mcr-1* gene is key in spreading colistin resistance among animal, environmental, and human reservoirs, enabling rapid horizontal transfer [31].

### Occurrence and distribution of ESBL-producing *E. coli*

In this study, 23.5% of *E. coli* isolates were identified as ESBL producers, a prevalence similar to that previously reported in healthy pigs in Thailand (19.2%) [4]. The detection rate of ESBL-producing *E. coli* was significantly higher in slaughterhouses (14.3%) than in fresh markets (9.2%) (p < 0.05), indicating greater contamination pressure at earlier stages of the pork production process rather than later stages.

Nevertheless, ESBL prevalence varied greatly when compared to other studies conducted in Thailand and other countries. For example, Boonyasiri *et al*. [32] reported ESBL prevalence of 33.3% in slaughterhouses and 61.5% in markets in eastern and northern Thailand, while Sornsenee *et al*. [33] documented ESBL-producing *E. coli* in minced chicken (79.17%), pork (43.75%), and beef (22.73%) in southern Thailand. Studies from other nations have also shown significant ESBL contamination in food animals and retail meat, including Singapore [34], Vietnam [35], South Korea [36], and Cameroon [37]. Such differences likely result from variations in sample types, sampling strategies, timing, farming practices, slaughterhouse hygiene, and antimicrobial use patterns.

The detection of ESBL-producing *E. coli* in both slaughterhouse and fresh-market samples indicates potential risks of transmission to consumers and environmental spread. Maintaining strict hygiene practices during and after slaughter is therefore essential to reduce fecal contamination and limit ESBL dissemination [33]. The presence of ESBL-producing *E. coli* on pork and cutting boards further suggests cross-contamination, likely from fecal sources, although molecular typing is needed to confirm transmission pathways.

### MDR among ESBL-producing isolates

An extremely high proportion (97.4%) of ESBL-producing *E. coli* isolates showed MDR, highlighting the serious clinical and public health concerns of ESBL phenotypes in pork production systems. Similar MDR rates have been reported in Thailand [33, 38], Vietnam [35], and Cameroon [37]. The high MDR frequency may result from extensive antimicrobial use in industrial pig farming, especially β-lactam antimicrobials used for disease prevention and growth promotion [39]. Such practices exert strong selective pressure, fostering the emergence and persistence of MDR organisms capable of spreading through food production systems to humans and the environment [40].

### β-lactamase gene profiles and their epidemiological significance

β-lactamase genes were found at significantly higher rates in ESBL-producing *E. coli* isolates from slaughterhouses compared to those from fresh markets (p < 0.05), indicating that slaughterhouses are a key control point for ESBL spread. Overall, 89.7% of ESBL-producing isolates carried at least one *bla* gene, with *bla*_TEM_ being the most common (66.7%). The TEM enzyme is the most widespread β-lactamase among gram-negative bacteria, providing resistance to penicillins and early-generation cephalosporins commonly used in pig farming [41, 42]. Similar high prevalence of *bla*_TEM_ has been reported in Thailand among healthy pigs and minced meat [33, 43].

The *bla*_CTX-M_ gene was found in 59% of ESBL-producing isolates, aligning with previous reports from Thailand [44] and other countries, including the United Kingdom, Germany, Tunisia, and Switzerland [45]. Notably, 35.9% of isolates carried more than one *bla* gene, providing evidence that ESBL-associated plasmids often contain multiple resistance determinants [46, 47]. These genetic configurations likely help maintain and spread β-lactam resistance throughout the pork production chain.

### Virulence gene distribution in *E. coli* isolates

Virulence genes were detected in only 3.2% of *E. coli* isolates, indicating a low prevalence of pathogenic *E. coli* within the study group. The identified virulence genes included *eaeA*, *lt*, and *stp*. The *eaeA* gene, which encodes intimin and is linked to EPEC and EHEC, was detected in both slaughterhouse and fresh-market samples and is associated with diarrheal illness in humans. The *lt* and *stp* genes, responsible for producing heat-labile and heat-stable enterotoxins in ETEC, respectively, were detected at very low levels [8, 48].

The low detection rate of virulence genes may be due to the age of the slaughtered pigs or to the lack of active infection at the time of sampling. Notably, the contrasting pattern seen in this study, high prevalence of AMR and ESBL production but low virulence gene detection, indicates that the selective pressures promoting AMR are different from those affecting the distribution of genes associated with pathogenicity.

### Limitations

This study has several limitations. First, using archived isolates collected during 2017–2018 limited the ability to directly trace or confirm cross-contamination pathways between slaughterhouses and individual fresh markets. Second, only a limited set of virulence genes was examined, which may underestimate the overall pathogenic potential of circulating *E. coli* strains. Third, molecular typing methods, such as whole-genome sequencing, were not performed due to financial constraints, thereby restricting the assessment of genetic relatedness and transmission dynamics along the pork production chain. Finally, environmental samples were not included, preventing the identification of potential AMR reservoirs within slaughterhouses and fresh markets. Despite these limitations, the study provides valuable baseline data to inform future research and guide targeted interventions to reduce AMR dissemination in pork production systems.

## CONCLUSION

This study revealed a significant burden of AMR throughout the pork production chain in central Thailand. Nearly all *E. coli* isolates showed resistance to at least one antimicrobial agent, with a large proportion classified as MDR. ESBL-producing *E. coli* were found frequently, with a notably higher prevalence in slaughterhouse samples compared to fresh-market samples, underscoring slaughterhouses as key upstream contamination sources. Most ESBL-producing isolates carried β-lactamase genes, mainly *bla*_TEM_ and *bla*_CTX-M_, and almost all exhibited MDR phenotypes. Conversely, virulence genes were rarely detected, suggesting that resistance traits are more widespread than pathogenic factors among the circulating *E. coli* populations.

The findings highlight the importance of slaughterhouses and fresh markets as key points in the spread of antimicrobial-resistant *E. coli* in the pork production chain. These settings allow fecal contamination, cross-contamination of carcasses, meat, and food-contact surfaces, and exposure of consumers. Improving hygiene and sanitation practices during slaughter, carcass processing, and retail handling is vital to decreasing the spread of resistant bacteria. Moreover, the high prevalence of MDR and ESBL-producing *E. coli* underscores the urgent need for cautious antimicrobial use in pig farming, especially by restricting the use of critically important antimicrobials. Coordinated surveillance across farms, slaughterhouses, and markets is essential to support evidence-based actions within a One Health approach.

A key strength of this study is the comprehensive assessment of AMR phenotypes, ESBL production, β-lactamase gene distribution, and virulence profiles in *E. coli* isolates collected from different stages of the pork production process. Including both slaughterhouse and fresh-market samples enabled direct comparison of upstream and downstream contamination points. Additionally, the relatively large number of isolates analyzed provides a solid baseline for understanding AMR dynamics in a region with intensive pig production.

Future research should include longitudinal sampling and environmental monitoring to better understand how antimicrobial-resistant *E. coli* spreads along the pork production process. Using molecular typing methods, such as whole-genome sequencing, would allow detailed analysis of strain relationships, resistance gene transfer, and potential contamination sources. Broadening the scope of virulence genes studied and adding data on antimicrobial use at the farm-level would enhance risk assessment and help develop targeted mitigation strategies.

In conclusion, this study emphasizes the widespread occurrence of AMR, MDR, and ESBL-producing *E. coli* throughout the pork production chain in central Thailand, with slaughterhouses identified as key points for the spread of resistance. Although pathogenic *E. coli* were found at low levels, the widespread presence of resistance factors presents a serious public health issue. Coordinated actions targeting antimicrobial stewardship, better hygiene practices, and ongoing surveillance are vital to prevent the transfer of AMR from pork production to humans and the environment.

## DATA AVAILABILITY

The supplementary data can be available from the corresponding author.

## AUTHORS’ CONTRIBUTIONS

OS and WP: Designed the study, collected samples, analyzed data, and drafted and revised the manuscript. YZ and CN: Designed the study, drafted, and revised the manuscript. AS, SW, AI, and RP: Analyzed data and drafted and revised the manuscript. All authors have read and approved the final version.
